# Parathyroid Hormone Signaling in Osteocytes

**DOI:** 10.1002/jbm4.10021

**Published:** 2017-11-10

**Authors:** Marc N Wein

**Affiliations:** ^1^ Endocrine Unit, Massachusetts General Hospital Harvard Medical School Boston MA USA

**Keywords:** OSTEOCYTE, PARATHYROID HORMONE, SCLEROSTIN, RANKL, BONE REMODELING

## Abstract

Osteocytes are the most abundant cell type in bone and play a central role in orchestrating skeletal remodeling, in part by producing paracrine‐acting factors that in turn influence osteoblast and osteoclast activity. Recent evidence has indicated that osteocytes are crucial cellular targets of parathyroid hormone (PTH). Here, we will review the cellular and molecular mechanisms through which PTH influences osteocyte function. Two well‐studied PTH target genes in osteocytes are SOST and receptor activator of NF‐κB ligand (RANKL). The molecular mechanisms through which PTH regulates expression of these two crucial target genes will be discussed. Beyond SOST and RANKL, PTH/PTH‐related peptide (PTHrP) signaling in osteocytes may directly influence the way osteocytes remodel their perilacunar environment to influence bone homeostasis in a cell‐autonomous manner. Here, I will highlight novel, additional mechanisms used by PTH and PTHrP to modulate bone homeostasis through effects in osteocytes. © 2017 The Authors. *JBMR Plus* is published by Wiley Periodicals, Inc. on behalf of the American Society for Bone and Mineral Research.

## Introduction

Parathyroid hormone (PTH) is the major endocrine regulator of extracellular calcium and phosphate levels.^1^ PTH and parathyroid hormone–related peptide (PTHrP) both signal through the same family B G‐protein‐coupled‐receptor (PTHR1, also known as PTH1R).^2^ Upon ligand binding, activated PTHR1 couples to multiple intracellular second messenger systems. Of these, Gαs‐dependent upregulation of cAMP plays a vital role in most of their biologic effects. However, intracellular coupling to other G proteins (Gαq, Gα12/13) and ß‐arrestin signaling exists as well.^3^ In addition to signaling via classic G‐protein–dependent mechanisms, PTH stimulates the formation of a ternary complex at the plasma membrane of PTH, the PTH/PTHrP receptor, and the WNT co‐receptor LRP6.^4^ When the activated PTH receptor binds to LRP6, this directly activates WNT signaling. Therefore, PTH/PTHrP directly engages multiple intracellular signaling mechanisms to exert effects on target cells in bone. Here we will focus on the intracellular mechanisms through which PTH/PTHrP influence osteocytes, the most abundant cell type in the skeleton.

Sustained hyperparathyroidism causes net loss of bone mass because of excessive stimulation of bone resorption but also increases osteoblast numbers and bone formation. In contrast, when injected once daily, intermittent PTH amino acids 1‐34 (teriparatide) treatment boosts bone mass, increases bone formation, and reduces fractures in individuals with osteoporosis.^5^, ^6^, ^7^ Recently, the PTHrP analog abaloparatide has also been shown to boost bone mass and reduce fractures in patients with osteoporosis when given by once‐daily subcutaneous injection.^8^ Currently, teriparatide and abaloparatide represent the only FDA‐approved osteoporosis medications that stimulate new bone formation.

It is well documented that parathyroid hormone promotes both bone formation and bone resorption; however, the cellular and molecular mechanisms through which this occurs are not fully understood. Osteocytes are crucial mediators of PTH action. Here, parathyroid hormone signaling in osteocytes will be reviewed in detail, with particular emphasis on the molecular mechanisms through which biologically relevant target genes are regulated.

Over the past three decades, multiple cellular mechanisms have been proposed to explain how intermittent PTH treatment increases bone formation by osteoblasts. Before diving into mechanisms of PTH signaling in osteocytes, important biologic effects in non‐osteocytic bone cells must be mentioned. PTH reduces osteoblast apoptosis.^9^ Direct effects of PTH on early cells in the osteoblast lineage have been proposed based on studies on cultured bone marrow–derived stromal cells (for example, Nishida and colleagues^10^) and, more recently, in vivo lineage tracing studies.^11^ PTH inhibits adipocyte differentiation of early stem cells in the osteoblast lineage^12^ and increases numbers of skeletal progenitors by reducing their apoptosis,^11^ providing additional mechanisms to explain how PTH influences differentiation of skeletal stem cells toward the osteoblast lineage. Rapid increases in osteoblast numbers found after iPTH treatment may be the result of direct conversion of previously quiescent bone lining cells into active osteoblasts.^13^ Non‐cell‐autonomous effects of PTH on osteoblast activity may also occur. For example, PTH‐induced osteoclastic bone resorption may liberate matrix growth factors that in turn recruit osteoblast progenitors to bone surfaces and stimulate their differentiation.^14^, ^15^ T lymphocytes in the bone marrow microenvironment respond to intermittent PTH by producing cytokines that stimulate osteoblast differentiation.^16^

Through effects on osteocytes, PTH regulates expression of osteocyte‐derived paracrine mediators that in turn influence bone remodeling, as will be reviewed here. These examples likely represent just the tip of the iceberg of how PTH utilizes multiple, complementary mechanisms to exert its profound influence on bone. Here, we will dive deeper into PTH signaling in osteocytes, highlighting current knowledge of the intracellular signaling pathways involved and pointing out major unresolved questions.

## Osteocytes as PTH Targets

The most abundant cell type in bone,^17^ osteocytes are former osteoblasts buried deeply within bone matrix. Osteocytes are strategically positioned to sense and respond to mechanical and hormonal cues. In doing so, osteocytes relay signals to osteoblasts and osteoclasts on bone surfaces that regulate bone homeostasis. Intensive research in the past 10 years has demonstrated that osteocytes are central to understanding skeletal responses to parathyroid hormone.

Before the recent explosion in knowledge regarding osteocytes came several clues suggesting that these cells respond to PTH. Osteocyte morphology is directly regulated by treatment with parathyroid extracts. This treatment causes dramatic changes in osteocyte appearance, including cellular retraction, mitochondrial engorgement, and cell death.^18^, ^19^ However, doses of PTH present in crude parathyroid extracts in these studies were exceptionally high, so it is possible that these changes may not reflect true physiologic (or pharmacologic) effects of parathyroid hormone. Because osteocytes are ideally poised to mobilize pools of calcium stored in bone, the concept of “osteocytic osteolysis” has been proposed as a mechanism through which PTH rapidly increases blood calcium levels.^20^, ^21^ More recently, further morphologic evidence supporting osteoclast‐independent mineral resorption has come to surface.^22^, ^23^ This phenomenon may be central to understanding changes associated with lactation, a physiologic state in which massive amounts of skeletal calcium must be liberated.^24^ Molecular mechanisms driving physiologic PTHrP‐driven perilacunar remodeling are reviewed below.

Mice in which the PTH receptor has been deleted using the best‐available osteocyte‐“specific” Cre lines reveal the physiologic role of PTH signaling in osteocytes during normal bone remodeling. Using the 9.6 kB DMP1‐Cre deleter strain,^25^ PTH receptor deletion causes mild increases in bone mass associated with reduced bone resorption,^26^ a phenotype reminiscent of what is observed in humans with hypoparathyroidism.^27^ Importantly, a similar low bone turnover phenotype in 8 kB DMP1‐Cre PTH receptor null mice is observed.^28^ Mice with osteocytes lacking PTH receptors have been used to ascertain the role of the osteocytic PTH receptor signaling in skeletal responses to intermittent and continuous PTH treatment. Significantly blunted/absent responses to intermittent PTH are observed when PTH receptors are not present in DMP1‐expressing cells using both Cre deleted strains.^26^, ^28^ Canonical PTH receptor signaling via Gsα/cAMP in osteoblast lineage cells is required for iPTH‐induced gains in bone mass.^29^ In contrast, apparently discordant results are observed with respect to responses to continuous hyperparathyroidism. In 9.6 kB DMP1‐Cre PTH receptor knockout mice, skeletal effects of continuous hyperparathyroidism (decreased cancellous bone mass, decreased cortical thickness, increased osteoid surface, increased osteoclasts) achieved via chronic PTH infusion were blunted/absent.^26^ In contrast, in 8 kB DMP1‐Cre PTH receptor knockout mice, skeletal effects of secondary hyperparathyroidism elicited by dietary calcium deficiency were completely normal.^28^

Several possibilities exist to account for these disparate findings. First, distinct models of continuous hyperparathyroidism were employed: The mechanisms that control bone loss in primary hyperparathyroidism (mimicked to some degree by PTH infusion) may be different than those operant in secondary hyperparathyroidism. For example, hypercalcemia might have direct effects on bone cells that interact with PTH signaling. Second, differences in experimental design (mouse age, duration of treatment, age at which treatment started, genetic background) exist between the two studies. Third, differences in patterns of Cre‐mediated deletion may exist comparing 9.6 kB and 8 kB DMP1‐Cre strains. Ultimately, a side‐by‐side comparison of how the two different Cre deleter strains “perform” with the same floxed gene of interest will be necessary to provide definitive proof for which strain is optimal for osteocyte‐“specific” gene deletion. Importantly, two recent studies using the 9.6 kB DMP1‐Cre strain in conjunction with a sensitive fluorescent reporter element demonstrate clear Cre‐dependent activity in non‐osteocyte cell types in and outside of bone.^30^, ^31^ To date, the same systematic analysis of 8 kB DMP1‐Cre mice using sensitive fluorescent reporter elements has not been reported. That being said, side‐by‐side comparison of 9.6 kB DMP1‐Cre and 8 kB DMP1‐Cre mice using a LacZ reporter allele has been performed. Appreciating technical limitations associated with beta galactosidase staining in adult bone sections, it does appear that both DMP1‐driven Cre strains show clear activity in matrix‐synthesizing osteoblasts along with osteocytes.^32^ SOST‐Cre mice^32^ represent an appealing model; however, these mice show high levels of Cre activity in hematopoietic cells, which limits their utility. Clearly, refined and improved tools are desperately needed to achieve osteocyte‐specific gene deletion in vivo.

## SOST: An Osteocyte‐Derived WNT Inhibitor Whose Expression Is Regulated by PTH

A critical observation that moved osteocyte biology forward came from human genetics. Individuals with sclerosteosis display very high bone mass and resistance to fractures. This rare Mendelian skeletal disease is caused by mutations in SOST, which encodes the protein sclerostin, an osteocyte‐specific secreted WNT inhibitor.^33^ Sclerostin is a tonic inhibitor of bone formation;^34^ therefore, a mechanism through which bone anabolic signals may trigger new osteoblast activity is by reducing SOST expression in osteocytes. Indeed, both skeletal loading^35^ and parathyroid hormone^36^, ^37^ rapidly reduce SOST levels in bone. This simple observation propelled osteocytes to the forefront in our thinking about how bone responds to PTH. In addition to SOST, osteocytes also are a major source of receptor activator of NF‐κB ligand (RANKL) in bone.^38^, ^39^ Osteocytic RANKL is upregulated by PTH and plays a vital role in PTH‐induced increases in bone resorption (see below).^40^, ^41^ Therefore, one attractive mechanism through which PTH signaling in osteocytes influences skeletal remodeling is by coordinated transcriptional regulation of paracrine mediators, including SOST and RANKL.

Multiple lines of mouse genetic evidence have highlighted the importance of PTH receptor signaling in osteocytes. First, artificially increasing PTH receptor signaling in osteocytes (achieved via transgenic expression of a constitutively active PTH receptor cDNA under the control of the 8 kB osteocyte‐enriched DMP1 promoter, DMP1‐caPTHR1 mice) leads to massive increases in bone mass and high turnover.^42^, ^43^ In these animals, constitutive PTH receptor signaling leads to reduced SOST expression and concomitant increases in WNT transcriptional output in osteocytes and osteoblasts. Accordingly, blocking WNT signaling via transgenic SOST overexpression or LRP5 deletion significantly blunts the phenotype in DMP1‐caPTHR1 animals. The contribution of increased osteoclast activity to this phenotype was addressed by treating DMP1‐caPTHR1 mice with alendronate. Interestingly, this pharmacologic manipulation reveals distinct, compartment‐specific effects of PTHR1 signaling in osteocytes: Alendronate reduces endocortical bone formation, has no effect on periosteal bone formation, and enhances cancellous bone mass in DMP1‐caPTHR1 animals.^44^ Future studies are needed to clarify the molecular mechanisms responsible for these compartment‐specific effects of PTH receptor signaling and osteoclasts.

Because SOST is downregulated by iPTH treatment and intact WNT signaling is required for mice to respond to iPTH,^45^ it has been of significant interest to determine if PTH‐induced SOST downregulation is required for iPTH‐induced bone anabolism. iPTH effects have been tested in two distinct SOST transgenic overexpressing strains. When a human SOST bacterial artificial chromosome (BAC) is used to overexpress sclerostin in bone, iPTH responses are significantly blunted.^46^ In contrast, when similar experiments are performed using a SOST transgene driven by the DMP1 promoter, iPTH treatment boosts bone mass in a normal manner.^28^ It is likely that differences between these two transgenic models account for discordant results. For example, the human SOST BAC (whose expression is inhibited by PTH) is probably expressed at higher levels at baseline than the DMP1‐SOST transgene. However, the fact that SOST‐deficient mice still boost bone mass in response to iPTH^46^ provides additional evidence that SOST downregulation is just one of many mechanisms used by PTH to stimulate WNT signaling and bone formation. Indeed, other cell types participate in the iPTH‐induced gains in bone mass. T lymphocytes produce WNT10B in response to intermittent PTH treatment, and this is important for gains in cancellous bone mass.^47^ Indeed, T‐cell–derived WNT10B and SOST downregulation work together as part of mechanism through which intermittent PTH treatment boosts bone mass.^48^

Recently, significant progress has been made toward understanding the molecular mechanisms within osteocytes through which PTH regulates target gene expression. Again, insights from human genetics have proved incredibly important in this area. Individuals with Van Buchem disease have high bone mass, resistance to fractures, and low levels of sclerostin. This rare monogenic disorder is caused by an intergenic deletion near the SOST gene such that includes a key downstream enhancer region.^49^ Early, pioneering work toward understanding the function of this downstream enhancer‐containing region^50^ ultimately identified a key binding site for the transcription factor MEF2C located 45 kB downstream of the SOST gene's transcription start site.^51^ Indeed, deletion of MEF2C in osteocytes^52^ or this MEF2 binding enhancer^53^ leads to low SOST expression and high bone mass.

Having identified that MEF2C is a crucial determinant of osteocytic SOST expression, an obvious question that emerges is whether PTH blocks MEF2C‐driven SOST expression. Studies using heterologous reporter systems suggested that cAMP signaling might regulate MEF2C activity in the setting of the +45 kB SOST enhancer.^51^, ^54^ In many biologic systems, upstream signals regulate MEF2 transcriptional activity via nucleo‐cytoplasmic shuttling of class IIa HDAC proteins, which serve as potent inhibitors of MEF2‐driven gene expression when present in the nucleus.^55^ PTHrP suppresses MEF2C‐driven chondrocyte hypertrophy^56^ by driving HDAC4 from the cytoplasm to the nucleus.^57^ In UMR106 osteocytic cells, PTH‐induced SOST suppression is associated with nuclear accumulation of HDAC5.^58^

Loss‐of‐function studies in conditionally immortalized Ocy454 cells (a PTH‐responsive murine osteocyte‐like cell line^59^, ^60^) and in mice reveal that deletion of both HDAC4 and HDAC5 is required to block PTH‐dependent SOST downregulation.^61^ Detailed studies into the signaling mechanisms upstream of PTH‐induced HDAC4/5 nuclear translocation have identified salt‐inducible kinase 2 (SIK2) as a crucial mediator of PTH signaling in osteocytes. SIK2 is a protein kinase A–regulated phosphoprotein; PKA‐mediated SIK2 phosphorylation reduces SIK2 cellular activity.^62^ Absent PKA phosphorylation, SIK2 tonically phosphorylates class IIa HDACs and promotes their cytoplasmic sequestration. As predicted by this model, small molecule SIK inhibitors^63^, ^64^, ^65^ such as YKL‐05‐099 reduce HDAC4/5 phosphorylation, promote their nuclear translocation, and reduce SOST expression in vitro and in vivo without increasing intracellular cAMP levels.^61^

**Figure 1:**
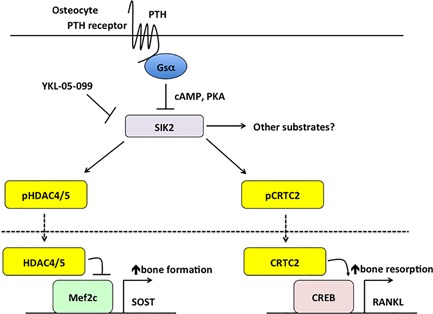
Schematic model demonstrating how PTH signaling leads to changes in SOST and RANKL expression in osteocytes. Salt‐inducible kinase 2 (SIK2) is a constitutively active kinase that normally phosphorylates substrates, including HDAC4/5 and CRTC2. When phosphorylated, these SIK2 substrates localize to the cytoplasm due to retention by 14‐3‐3 chaperone proteins. PTH induces protein kinase A activation, which phosphorylates and inhibits SIK2 and leads to reductions in SIK substrate phosphorylation and their subsequent nuclear translocation. In the nucleus, HDAC4/5 inhibit MEF2C‐driven SOST expression, and CRTC2 potentiates CREB‐driven RANKL transcription. Because PTH inhibits SIK2 action, small molecule SIK inhibitors such as YKL‐05‐099 mimic the effects of PTH in a cyclic AMP‐independent manner. Future studies are needed to assess whether additional PTH‐regulated SIK substrates exist and to determine the role of PTH‐dependent SIK2‐independent pathways in osteocytic responses to parathyroid hormone. It is not currently known if this pathway also participates in PTH/PTHrP‐driven osteocytic perilacunar remodeling.

Surprisingly, small molecule SIK inhibitors mimic effects of PTH beyond SOST regulation. By reducing CRTC2 phosphorylation, these agents induce RANKL expression (see below). At the transcriptomic level, approximately 32% of PTH‐regulated genes are co‐regulated by SIK inhibitor treatment. Although HDAC4/5‐deficient mice show normal bone anabolic responses to iPTH, YKL‐05‐099 treatment boosts bone formation and bone mass in vivo.^61^ These studies highlight the importance of SIK2‐regulated phosphoproteins (such as HDAC4/5 and CRTC2) in mediating the intracellular effects of PTH in osteocytes and identify SIK inhibition as a promising strategy to mimic skeletal effects of PTH (Fig. 1). Several major questions remain regarding the role of salt‐inducible kinases downstream of PTH receptor signaling. First, it is not known whether these kinases participate in PTH responses in non‐osteocyte PTH target cells. Second, we do not currently know if additional PTH‐regulated SIK2 substrates in osteocytes exist. Third, genetic evidence that salt‐inducible kinases deletion leads to skeletal phenotype similar to constitutively active PTH receptor overexpression remains to be determined. Fourth, PKA‐mediated inhibition of SIK activity appears to be a conserved feature downstream of multiple Gαs‐linked hormonal signaling modules.^62^, ^66^, ^67^, ^68^ Therefore, additional studies are required to understand how specific signaling outputs are achieved downstream of PTH/SIK pathway engagement in osteocytes. Finally, additional studies are needed to further explore the utility of small molecule SIK inhibitors (like YKL‐05‐099) as potential therapeutic agents for osteoporosis treatment.

## Mechanisms Controlling PTH‐Induced RANKL Upregulation

A well‐characterized mechanism through which PTH induces osteoclastic bone resorption is via upregulation of RANKL.^69^ Two lines of evidence point to an important role for osteocytes as a major source of RANKL during normal bone remodeling. First, osteoblast depletion strategies largely preserve osteoclasts and RANKL levels.^70^, ^71^ Second, conditional deletion of RANKL using the best‐available osteocyte‐“specific” Cre deleter strains leads to reduced osteoclast numbers and high bone mass.^32^, ^38^, ^39^ Future studies are needed to clarify whether soluble or membrane‐bound RANKL^72^, ^73^ participates in how osteocytes regulate remodeling on bone surfaces. An added layer of complexity emerges from the observation that sclerostin may promote RANKL expression by osteocytes.^74^

Multiple external cues modulate RANKL expression in bone cells, including parathyroid hormone 1,25‐dihydroxyvitamin D and various pro‐inflammatory cytokines.^75^, ^76^ Significant progress has been made in recent years toward understanding the molecular mechanisms through which RANKL transcription is regulated. Importantly, the majority of these mechanistic studies have been performed in osteoblast‐like cell lines. Therefore, it is possible that some of the lessons learned may not necessarily apply to osteocytes. Cyclic AMP signaling downstream of the PTH receptor induces RANKL expression via distinct enhancer located ∼75 kB upstream of the gene's transcription start site.^77^, ^78^, ^79^ Targeted deletion of this enhancer reduces basal and PTH‐induced RANKL expression in bone, leading to hypoparathyroidism‐like skeletal changes.^80^ Interestingly, deletion of this enhancer is sufficient to blunt skeletal RANKL upregulation stimulated by dietary calcium deficiency and lactation but insufficient to block bone loss associated with these conditions.^81^ These results indicate that additional mechanisms can compensate to promote skeletal calcium liberation when RANKL upregulation cannot occur. Multiple possibilities may explain this fascinating paradox. First, RANKL expression by other cell types (lymphocytes in bone marrow, which were not included in the RNA analyses performed in these studies) might substitute for skeletal sources of RANKL to promote bone loss when this enhancer is deleted. Second, PTH‐dependent RANKL‐independent mechanisms that drive osteocytic perilacunar remodeling (see below) might compensate when RANKL cannot be upregulated because of enhancer deletion. Third, secondary hyperparathyroidism massively increases serum levels of 1,25‐dihydroxyvitamin D. It is possible that activated vitamin D also might have direct effects on osteocytes that promote RANKL‐independent liberation of calcium stores.

In addition to the −75 kB “D5” RANKL enhancer, multiple additional enhancers have been identified that contribute to cell type–specific RANKL regulation.^82^ For example, although the −75 kB D5 enhancer appears to be most important in bone lineage cells, a distinct regulatory region located ∼200 kB upstream of the human RANKL gene is involved in T lymphocyte RANKL expression.^83^ It is quite interesting that several bone mineral density (BMD)‐associated RANKL variants map to these T‐cell enhancer regions.^84^ Corresponding murine enhancer regions are required for T‐cell RANKL expression during inflammatory conditions in vivo.^85^ Finally, recent studies have identified a second “D2” RANKL enhancer ∼23 kB upstream of the gene's start site whose deletion largely phenocopies mice lacking the −75 kB D5 enhancer.^86^ Future studies will be needed to clarify the relationship between the D2 and D5 enhancers in basal and PTH‐stimulated osteocytic RANKL regulation.

Although it has been long appreciated that PTH‐induced RANKL upregulation requires cAMP/protein kinase A signaling,^76^, ^87^ the nature of the downstream signaling steps has remained somewhat more mysterious. The transcription factor CREB is a well‐known PKA substrate at serine 133,^88^ and phospho‐CREB RANKL enhancer binding is observed in response to PTH and other cAMP‐inducing signals.^78^ However, CREB deletion in osteoblast/osteocyte lineage cells does not affect osteoclast numbers or RANKL expression in vivo.^89^ Remarkably, another bZIP family transcription factor, ATF4, is required for RANKL upregulation in osteoblastic cells in response to ß2‐adrenergic (but not PTH) signaling events by binding to the proximal RANKL promoter.^90^ Therefore, multiple bZIP family transcription factors likely participate in distinct ligand‐induced RANKL upregulation pathways. Future studies will be needed to understand how two ligands (PTH and isoproterenol) that signal through Gαs‐linked G‐protein‐coupled receptors regulate RANKL through distinct downstream mechanisms.

Cyclic AMP‐regulated transcriptional co‐activators (CRTC) family transcriptional co‐activators^91^ provide an additional layer of regulation in PTH‐induced RANKL induction. CRTC proteins are latent co‐activators of CREB (and other bZIP family transcription factors) that shuttle from the cytoplasm to the nucleus in response to upstream signals. In osteocytes, CRTC2 is tonically phosphorylated by SIK2 and therefore sequestered in the cytoplasm. Upon PKA‐mediated SIK2 phosphorylation and inactivation, CRTC2 phosphorylation levels decrease, and this protein is able to translocate from the cytoplasm to the nucleus. In the nucleus, CRTC2 associates with the aforementioned D2 and D5 RANKL enhancer regions and is required for PTH‐induced RANKL upregulation in Ocy454 cells.^61^ Whether CRTC2 functions with CREB or with other bZIP factors remains unknown, as is the role of CRTC2 in osteocyte function in vivo.

## Expanding the Repertoire of PTH/PTHrP Actions in Osteocytes

Many PTH‐regulated genes in osteocytes are *not* regulated in a SIK2‐dependent manner. Therefore, additional intracellular signaling nodes downstream of the PTH receptor must exist. Nascent polypeptide‐associated complex and co‐regulator alpha (αNAC) is another PKA substrate that shuttles from the cytoplasm to the nucleus upon phosphorylation.^92^ In the nucleus, αNAC associates with bZIP family transcription factors and enhances their activity.^93^ LRP6 is one such PTH‐induced aNAC target gene;^94^ a WNT co‐receptor, LRP6 is required for iPTH‐induced bone anabolism.^95^, ^96^ Therefore, it is likely that PTH employs complementary intra‐ and intercellular mechanisms in osteocytes to stimulate WNT signaling.

Beyond these focused studies on candidate signaling molecules, several groups have recently performed unbiased approaches to delineate genes regulated by PTH in osteocytes. Like Ocy454 cells, IDG‐SW3 cells are a conditionally immortalized murine osteocyte‐like cell line.^97^ RNA‐seq analysis of these cells over the course of their differentiation and in response to PTH has been completed.^98^, ^99^ Interestingly, transcriptional effects of PTH in this cell type are largely similar to those of vitamin D. PTH treatment of IDG‐SW3 cells appears to cause them to revert to a less mature, more osteoblast‐like phenotype. When mature IDG‐SW3 cells are treated with PTH, striking morphologic changes are observed, including fewer dendritic extensions and increased motility.^100^ Although the mechanistic basis for these phenomena remain incompletely understood, effects on calcium channel gene expression may contribute. PTH increases expression of L‐type (osteoblastic) calcium channels and reduces expression of T‐type (osteocytic) channels. L‐type calcium channels are partially responsible for PTH‐induced changes in osteocyte morphology, as evidenced by pharmacologic experiments with diltiazem.^100^

Rapid PTH‐induced changes in osteocyte morphology may provide an important mechanistic clue into how PTH and PTHrP quickly liberate skeletal calcium stores during normal physiologic stresses such as calcium deficiency and lactation.^101^ As described above, historic studies in which rodents were administered extremely high doses of parathyroid hormone in the form of parathyroid abstract led to obvious overt changes in osteocyte morphology. More recently, the physiologic relevance of these seminal observations has been tested in the setting of perilacunar remodeling. Osteocytes can remove bone matrix during lactation by reversible remodeling of the perilacunar/canalicular network. Surprisingly, osteocytes in lactating mice express cathepsin K and TRAP, genes traditionally thought to be restricted to osteoclasts. Infusion of PTHrP, whose levels are normally high during lactation,^102^ mimics many of these changes. Mice lacking PTHrP in the mammary gland fail to lose bone during lactation,^103^ highlighting the importance of breast‐derived PTHrP as a physiologic signal for lactation‐induced bone remodeling. Furthermore, osteocytes lacking PTH receptors fail to undergo perilacunar remodeling during lactation.^101^ In addition to upregulating cathepsin K and TRAP, PTHrP promotes osteocytic expression of ATP6V0D2, a vacuolar ATPase associated with osteoclastic bone resorption. Indeed, lactating calcium‐deficient mice show reduced perilacunar pH, as assessed using a novel GFP‐based reporter system.^104^ PTH/PTHrP‐dependent regulation of perilacunar pH may represent a rapid mechanism for osteocytes to liberate readily accessible pools of calcium. Future studies will be needed to assess the relative contribution of this pathway versus osteoclastic bone resorption and to precisely define the molecular signature associated with physiologic and pathologic osteocytic perilacunar remodeling.

Recent genetic evidence has confirmed a role for PTHrP expression in 9.6 kB DMP1‐Cre‐expressing cells in physiologic bone remodeling.^105^ Mice in which PTHrP (encoded by the PTHLH gene) is deleted using this Cre driver show low cancellous bone mass, reduced osteoblast activity, high local sclerostin levels, and reduced cortical bone strength. Interestingly, Ocy454 osteocyte‐like cells express endogenous PTHLH transcripts, and loss‐of‐function studies demonstrate that PTHLH tonically controls expression of SOST, other osteocyte marker genes, and basal cAMP levels in these cells. Interestingly, DMP1‐Cre deletion of PTHrP does not perturb osteoclast numbers or RANKL levels, indicating that PTHrP‐stimulated RANKL expression is dispensable during normal physiology.^105^ The relative contribution of bone‐ versus breast‐derived PTHrP^103^ during lactation remains to be determined. In addition, future studies are needed to address whether changes in locally produced PTHrP contribute to responses to mechanical loading, especially given the interesting observation that the PTHR1 is required for optimal bone anabolic responses to this stimulus.^28^

Based on recent clinical data indicating beneficial effects of the PTHrP analog abaloparatide at predominantly cortical sites,^8^ it will be important to study differences between PTH and PTHrP in inducing osteocytic gene expression and perilacunar remodeling. To date, very little is known about molecular differences between these two PTHR1 ligands. Using overexpressed receptors in heterologous systems, it has been noted that PTH preferentially binds to the R0 PTHR1 conformation, whereas abaloparatide favors the transient RG configuration.^106^ This difference in receptor engagement may contribute to biologic differences in signaling outputs, although this appealing hypothesis remains to be tested at the level of endogenous receptors in bone cells. Importantly, microarray‐based expression profiling in osteoblastic cells (Kusa 4b10, UMR106, and calvarial osteoblasts) previously revealed no obvious differences comparing PTH and PTHrP.^107^ To date, studies examining the receptor conformation preferences of full‐length PTHrP have not been performed.

## Summary

Basic, translational, and clinical research over the past three decades has identified parathyroid hormone and its analogs as important bone anabolic drugs for osteoporosis and illuminated many of the cellular and molecular mechanisms through which these agents regulate bone remodeling. As discussed, there is no one single mechanism to explain how intermittent PTH treatment increases bone formation and bone mass. Instead, multiple complementary mechanisms coordinately explain the potent effects of this hormone, which evolved as the central regulator of calcium metabolism, on skeletal physiology.

Major gaps and unanswered questions currently exist in our understanding of how the mechanisms in osteocytes described work together to explain biologic effects of the hormone. Although it is obvious that there is more to PTH‐induced bone anabolism than SOST downregulation, the requirement for intact WNT signaling for PTH‐induced gains in bone mass is quite clear.^45^ Therefore, which molecular (cell‐intrinsic and cell‐extrinsic) mechanisms are most important to explain cross‐talk between PTH and WNT signaling? What is the relative role of PTH/PTHrP‐driven osteocytic osteolysis versus PTH‐driven RANKL‐dependent osteoclastogenesis in calcium metabolism? Do differences exist at the molecular level between osteocytic responses to pharmacologic doses of intermittent teriparatide and abaloparatide? Which intracellular arms and target genes of the osteocytic PTH responses are most important for its ability to induce bone formation? Finally, what is the relationship between the effects of PTH on mineral metabolism in the kidney^108^ and its effects on the skeleton? Ultimately, a thorough understanding of how intermittent PTH therapy affects bone will be necessary to design new and improved future osteoporosis treatments.

## Disclosures

The author states that he has no conflicts of interest.
